# Risk factors for allergic rhinitis in children: an umbrella review of systematic review and meta-analysis

**DOI:** 10.3389/fimmu.2026.1849271

**Published:** 2026-09-07

**Authors:** Yuzhu Dou, Jinyi Li, Xueyan Qiu, Pengchen Wu, Yaqi Wang, Mei Chen, Zhengjiu Cui, Fei Luo, Siming Zhai, Tao Li, Qigang Dai, Bin Yuan

**Affiliations:** 1 Department of Pediatrics, Affiliated Hospital of Nanjing University of Chinese Medicine, Nanjing, China; 2 Department of Pediatrics, The First Affiliated Hospital of Zhejiang Chinese Medical University, Hangzhou, China; 3 Department of Pediatrics, Wuxi Affiliated Hospital of Nanjing University of Chinese Medicine, Wuxi, China; 4 Department of Pediatrics, Wuxi Hospital of Traditional Chinese Medicine, Wuxi, China

**Keywords:** allergic rhinitis, children, meta-analysis, risk factors, umbrella review

## Abstract

**Background:**

This study evaluates the quality of study design, potential biases, and evidence strength for childhood risk factors associated with allergic rhinitis (AR), while summarizing the key identified factors.

**Methods:**

The protocol for this review was registered in PROSPERO (CRD420251248436). Relevant literature was systematically retrieved from PubMed, Web of Science, Embase, and the Cochrane Database of Systematic Reviews, covering the period from inception to May 2025. Methodological quality and evidence certainty were independently evaluated using AMSTAR and GRADE.

**Results:**

This review identified 75 risk factors: 22 were linked to a higher risk of AR in children, 7 were protective, and 46 showed insufficient evidence to determine an association. Overall, evidence quality was low or very low, with only one factor supported by moderate-quality evidence. Key findings indicate that early-life antibiotic use, cesarean section, smoking during pregnancy, air pollution, ADHD diagnosis, ASD diagnosis, Kawasaki disease and maternal OCP exposure were associated with higher risk, while pet exposure, raw milk and fish consumption, birth order ≥ 2, and number of siblings ≥ 2 are associated with reduced risk.

**Conclusion:**

This first umbrella review systematically compiles all available meta-analytic data on pediatric AR risk factors, offering a provisional evidence map that prioritizes candidate modifiable targets for future research. Earlylife food sensitization is the only factor with moderate certainty. However, the generally low certainty of evidence underscores an urgent need for more rigorous and standardized primary studies and meta-analyses to strengthen the foundation for clinical and public health interventions.

**Systematic review registration:**

https://www.crd.york.ac.uk/PROSPERO/, identifier CRD420251248436.

## 1 Introduction

Allergic rhinitis (AR) is an inflammatory condition involving the nasal mucosa, in which patients commonly experience nasal itching, sneezing, rhinorrhoea, and nasal blockage (1). The condition involves both early- and late-phase hypersensitivity reactions and is classified as intermittent (seasonal) or persistent (perennial) AR (2). With an estimated 500 million individuals affected globally, AR is among the most frequently occurring chronic diseases and the leading chronic condition among children in the United States, thereby posing a substantial economic and health burden (3, 4). In pediatric patients, the effects of AR on quality of life are often less obvious than in adults and may manifest as fatigue, reduced attention span, and impairments in learning and memory, which are frequently overlooked or misinterpreted by parents as behavioral problems (5). A transition in the management of AR, from controlling symptoms to precision prevention grounded in addressing potentially modifiable correlates, is crucial to mitigate the disease’s dual burden on child health and socioeconomic outcomes.

Multiple meta-analyses have identified various risk factors associated with AR in children, including parental genetic background, pets, caesarean section, damp environments, and other comorbid conditions (6–9). Additionally, immune-associated biomarkers such as CST1 and SERPINB4, gut microbiota, and epigenetic modifications in dendritic cells have been shown to be essential for the core mechanisms of AR pathogenesis in children (10–12). However, the existing evidence remains fragmented and inconsistent. First, studies of the same risk factor frequently yield heterogeneous results. For example, whereas early investigations into pet exposure largely emphasized harmful effects (13), more recent, large-scale analyses suggest a protective effect (14). Such discrepancies may be attributed to differences in study design, population characteristics, and exposure definitions. Second, current systematic reviews are often limited to specific risk-factor categories, thereby lacking a comprehensive synthesis of risk factors for AR as a whole. Furthermore, with ongoing methodological advances, the evidence base requires a contemporary evaluation of its quality and robustness, particularly through a rigorous assessment of potential publication bias and sources of heterogeneity.

We conducted a comprehensive synthesis of existing meta-analyses to integrate evidence on risk factors for AR in children. To our knowledge, no previous umbrella review has systematically evaluated the full spectrum of factors associated with childhood AR. The unique contribution of this study lies in: (1) for the first time, an umbrella review method was adopted to systematically integrate the evidence of 75 risk factors from 56 Meta-analyses, covering the entire life cycle exposure from prenatal to childhood; (2) using the AMSTAR and GRADE tools, a unified assessment of the methodological quality and evidence certainty of all the evidence was conducted, revealing the weak points of the existing evidence base; (3) by identifying the evidence gaps and sources of heterogeneity, clear direction guidance for future research was provided, including the need for standardized exposure definitions, prospective cohort designs, and adequate control of confounding factors; (4) a hierarchical prevention framework based on evidence grades for pediatric AR was proposed, distinguishing high-priority intervention targets, targets requiring individualized assessment, and directions for further research.

## 2 Methods and analysis

### 2.1 Design and registration

A systematic review of the literature was performed, with data extraction and analysis of risk factors for allergic rhinitis in children conducted in accordance with the PRISMA guidelines (15). This umbrella review followed the methodological framework outlined in the Joanna Briggs Institute Manual for Evidence Synthesis of Umbrella Reviews (16, 17). In addition, the review protocol was registered in PROSPERO (ID: CRD420251248436, https://www.crd.york.ac.uk/PROSPERO/).

### 2.2 Eligibility criteria

Risk factors for AR in children across different ethnicities, geographic regions, sexes, and study settings were considered. When more than one meta-analysis addressing the same risk factor was published more than 24 months apart, the most recent publication was selected. If multiple meta-analyses were released within a 24-month interval, priority was given to the study including the largest number of prospective cohort studies; when this criterion was equal, the meta-analysis with the higher AMSTAR score was chosen (18, 19). In cases where the most recent meta-analysis did not report a dose–response analysis but another did, both were included for data extraction. Meta-analyses published in languages other than English, as well as studies based on animal experiments or cell culture models, were excluded.

### 2.3 Sources

We performed a thorough search of PubMed, Web of Science, Embase, and the Cochrane Database of Systematic Reviews, including all available records up to May 2025. This search focused on meta-analyses encompassing both interventional and observational studies. To ensure comprehensive coverage, we conducted a manual examination of the reference lists of the selected meta-analyses to uncover any additional pertinent studies.

### 2.4 Search strategy

The search strategy included the following terms: (((“Risk Factors”[Mesh]) OR ((((((((((((Risk Factor) OR (Population at Risk)) OR (Populations at Risk)) OR (Risk Scores)) OR (Risk Score)) OR (Risk Factor Scores)) OR (Risk Factor Score)) OR (Health Correlates)) OR (Social Risk Factors)) OR (Social Risk Factor)) OR (Risk)) OR (Incidence))) AND ((“Rhinitis, Allergic”[Mesh]) OR ((Allergic Rhinitides) OR (Allergic Rhinitis)))) AND (systematic review OR meta-analysis) (**Supplementary Table 1**) (20).

### 2.5 Assessment of methodological quality

Two authors independently evaluated the methodological robustness of each meta-analysis using AMSTAR, a recognized tool for appraising systematic reviews and meta-analyses (18, 21). Additionally, the GRADE system was applied to assess the quality of evidence for each health outcome, classifying it into ‘high’, ‘moderate’, ‘low’ or ‘very low’, to reflect the evidence’s reliability (22).

### 2.6 Data extraction

Data extraction from each eligible study was conducted independently by two authors, focusing on the following information: 1) author name, 2) publication year, 3) risk factors evaluated, 4) study population, 5) number of included studies, 6) total participants and cases, 7) study design (randomized controlled trial [RCT], cross-sectional, case-control or cohort), 8) outcomes, and 9) effect size estimates (RR, OR, or HR) with 95% confidence intervals (CIs). Additional details were also extracted, including the meta-analytic model (random or fixed effects), heterogeneity statistics (I^2^ and Cochran’s Q-test), and assessment of small-study bias (Egger’s test, Begg’s test, and funnel plot). Any disagreements between the authors were resolved through consultation with a third author.

### 2.7 Data summary

Relative risks (RR), odds ratios (OR), or hazard ratios (HR) with 95% CIs were re-estimated using either fixed-effects or random-effects models, depending on the characteristics of the data. Heterogeneity was assessed using I² statistics and Cochran’s Q test, and small-study effects were evaluated with Egger’s or Begg’s tests for meta-analyses containing more than 10 studies (23–25). For risk factors rated as high or moderate quality, sensitivity analyses were conducted, where applicable, to examine the impact of individual studies on the overall evidence. A P value < 0.10 was considered indicative of significant heterogeneity, while a P value < 0.05 was deemed important for other tests. Evidence synthesis was conducted using Review Manager (version 5.4, Cochrane Collaboration, Oxford, UK), and Egger’s and Begg’s tests, along with sensitivity analyses, were performed using Stata (version 15.1).

## 3 Results

### 3.1 Characteristics of meta-analyses

**Figure 1:**
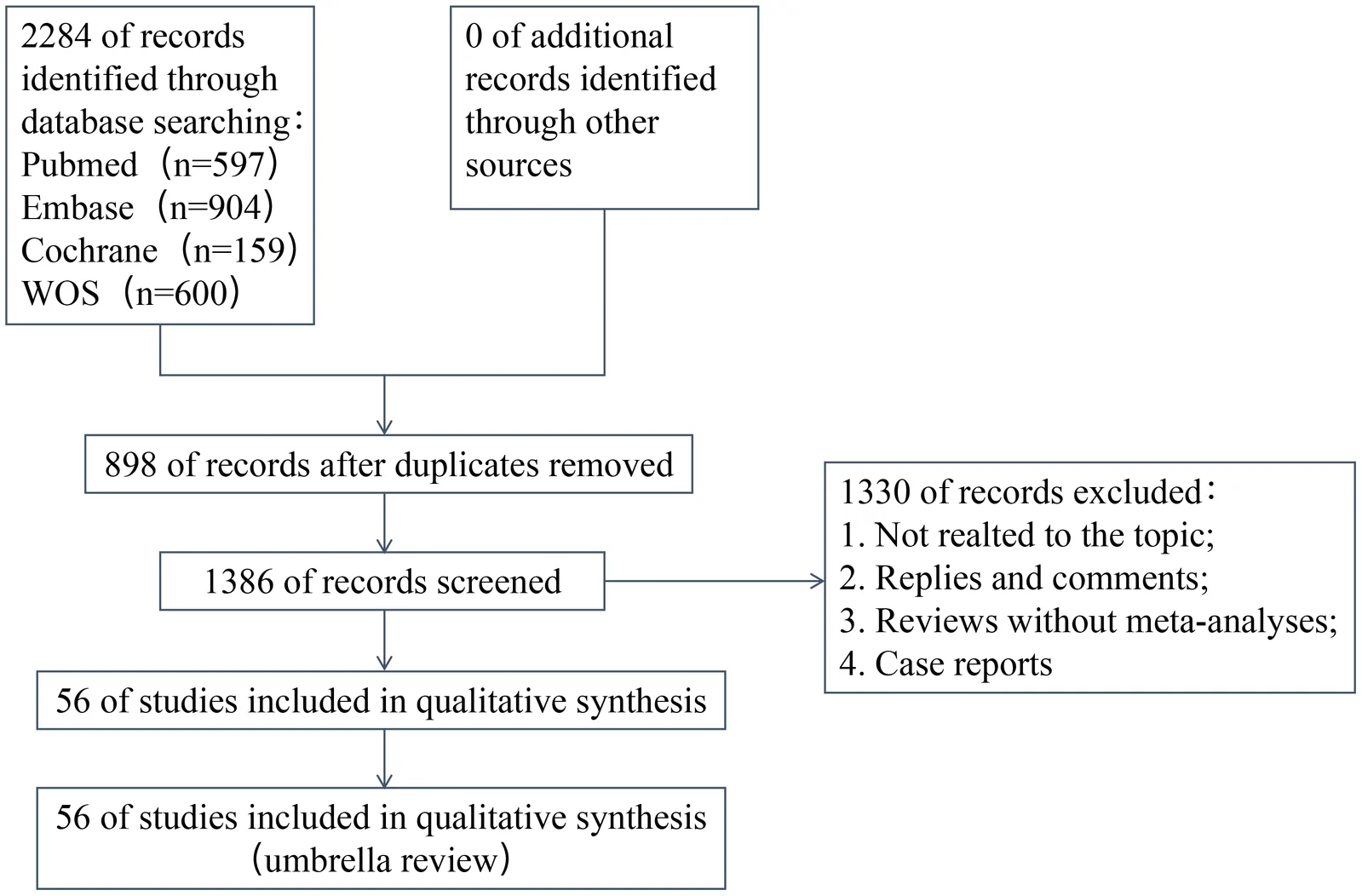
Flow chart of the systematic search and selection process.

**Figure 2:**
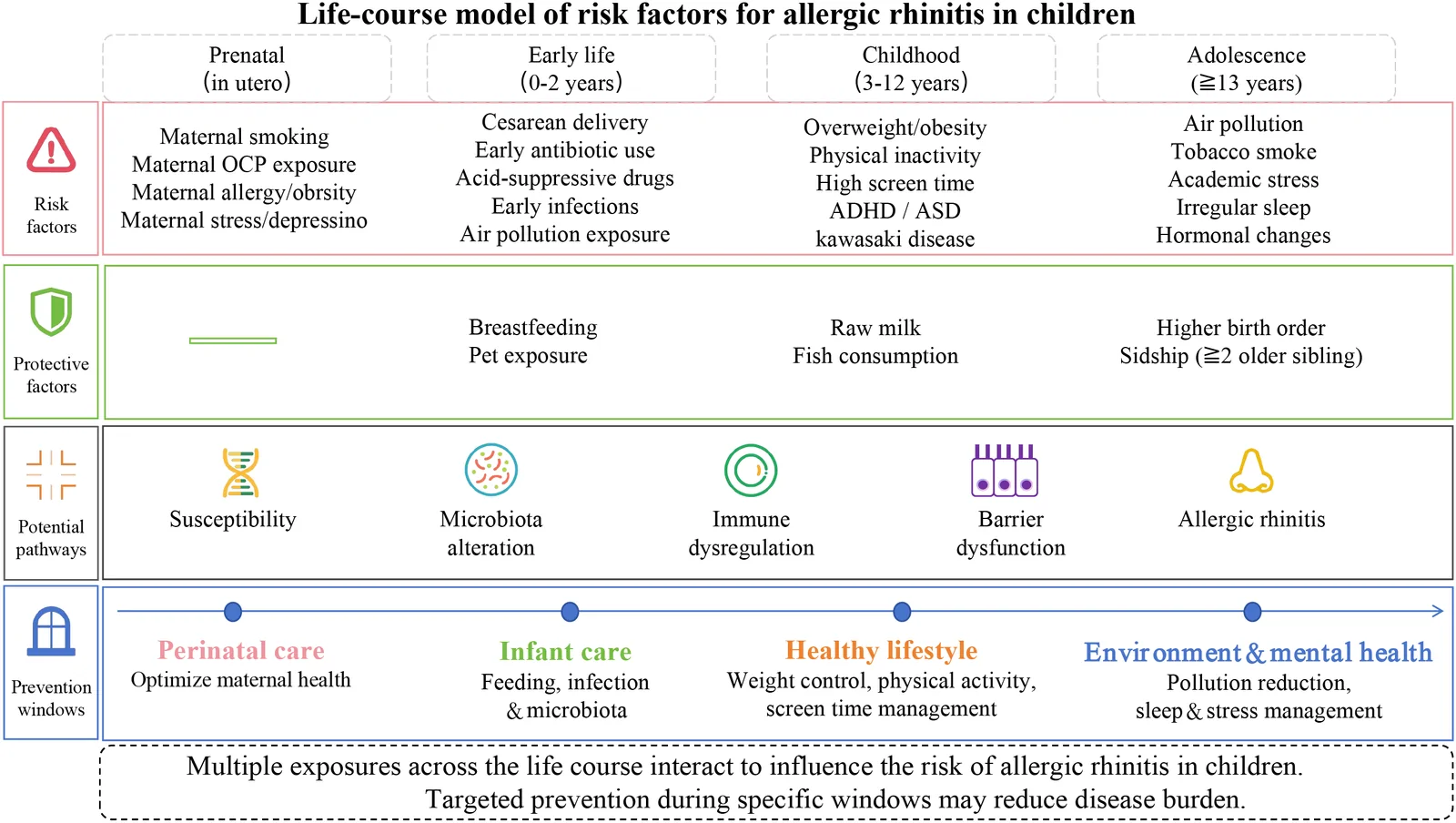
Forest plots of significant-associated risk factors for allergic rhinitis in children.

**Figure 1** illustrates the flowchart detailing the literature search and selection process. A total of 2,284 unique articles were identified through the systematic literature search. Based on the inclusion criteria, 56 meta-analyses were included in the review (26–65). We extracted 75 unique risk factors (**Table 1**). Of these, 22 risk factors were identified as deleterious, while seven were considered beneficial. Meanwhile, **Figure 2** presents a forest plot of the significant-associated risk factors for childhood AR. Detailed AMSTAR scores for each result are provided in **Supplementary Table 2**. The median AMSTAR score across all risk factors was 8 (range 7-10). Detailed GRADE evaluation results are presented in **Supplementary Table 3**. According to the GRADE evaluation criteria, most findings were categorized as either low or very low in quality. Only one risk factors was rated as moderate quality, and the majority of risk factors for AR in children were related to perinatal factors, early exposure, and environmental factors. Sensitivity analyses of outcomes rated as moderate quality confirmed the direction and significance of the associations, indicating robust findings. Among the reasons for downgrade, the risk of bias is the most common dimension for downgrade, and almost all observational studies have been downgraded due to limitations in the study design; inconsistency (i.e., high heterogeneity) is the second most common reason for downgrade, especially in factors such as pet exposure, ADHD, and KD, which have extremely high heterogeneity; imprecision is also an important downgrade factor in some analyses based on a small number of original studies or small samples.

**Table 1:** Risk factors for allergic rhinitis in children.

Population	Risk factors	Assessed with	Included MA	References	Sample size case/total	MA metric	Estimates [95% CI]	No. of studies T/R/C/P	Effects model	I2; Q test P value	Egger test P value	AMSTAR	GRADE
Significant associations													
AR children >2-12y	Early life food sensitization	With vs. without	Alduraywish 2015	37	2184/4386	OR	3.06 [1.90 to 4.94]	4/0/4/0	Random	38.0%; 0.184	NA	8	Moderate
AR Chinese children	PM_10_	Highest vs. lowest	Zhang 2020	38	NA/129597	OR	1.02 [1.01 to 1.03]	10/0/5/5	Fixed	48%; < 0.04	NA	8	Low
AR children	Exposed to pets	Any vs. none	Qiu 2024 (44)	44	3004/11751	OR	0.25 [0.12 to 0.39]	8/0/8/0	Random	99.8%; 0.000	NA	9	Low
AR children	Early-life antibiotic use	antibiotic vs. no medication	Liu 2022 (35)	35	NA/74677	OR	3.38 [3.21 to 3.56]	13/0/8/5	Random	60.7%; 0.002	NA	8	Low
AR children	Early-life antibiotic use	antibiotic vs. no medication	Liu 2022 (35)	35	NA/146280	HR	5.71 [5.54 to 5.88]	2/0/0/2	Random	92.3%; 0.000	NA	8	Low
AR Chinese children	NO_2_	Highest vs. lowest	Zhang 2020	38	NA/119637	OR	1.11 [1.05 to 1.18]	9/0/4/5	Random	80%; < 0.00001	NA	8	Very low
AR Chinese children	SO_2_	Highest vs. lowest	Zhang 2020	38	NA/30513	OR	1.03 [1.01 to 1.05]	7/0/4/3	Fixed	0%; 0.58	NA	8	Very low
AR Chinese children	PM_2.5_	Highest vs. lowest	Zhang 2020	38	NA/92722	OR	1.15 [1.03 to 1.29]	4/0/1/3	Random	83%; 0.0006	NA	8	Very low
AR children	Phthalates and their metabolites exposure in maternal urine	Highest vs. lowest	Oh 2024 (39)	39	NA/3588	OR	1.041 [1.003 to 1.081]	3/0/3/0	Random	0%; 0.263	0.81992	8	Very low
AR children	Maternal OCP exposure	With vs. without	Bai 2020 (32)	32	2439/5386	OR	1.34 [1.07 to 1.68]	4/0/1/3	Random	38.8%; 0.162	NA	7	Very low
AR children	Raw milk consumption in the first years of life	Highest vs. lowest	Brick 2019	26	NA/64685	OR	0.68 [0.57 to 0.82]	6/0/3/3	Random	0%;NA	NA	7	Very low
AR children	Current raw milk consumption	Highest vs. lowest	Brick 2019	26	NA/64685	OR	0.68 [0.54 to 0.85]	4/0/1/3	Random	NA	NA	7	Very low
AR children	Prenatal smoke exposure	Any vs. none	Li 2022 (33)	33	NA	OR	1.12 [1.04 to 1.21]	6/0/6/0	Random	0%; 0.570	NA	7	Very low
AR children	Postpartum smoke exposure	Any vs. none	Li 2022 (33)	33	NA	OR	1.19 [1.03 to 1.39]	8/0/8/0	Random	76.8%; 0.000	NA	7	Very low
AR children ≤ 4 y	Fish introduction before age 6 to 12 months	With vs. without	Ierodiakonou 2016 (27)	27	717/10311	OR	0.59 [0.40 to 0.87]	3/0/3/0	Random	59.2%; 0.09	NA	8	Very low
AR children	High intake of fish during pregnancy or infancy	With vs. without	Zhang 2016	28	NA/9987	RR	0.54 [0.36 to 0.81]	3/0/3/0	Random	74%; 0.02	NA	8	Very low
AR children	Maternal exposure to smoking during pregnancy	With vs. without	Zhou 2021 (34)	34	NA/1149879	OR	1.13 [1.02 to 1.26]	16/0/7/9	Random	81%; < 0.00001	NA	8	Very low
AR children	Infection during pregnancy	high vs. low or none	Chen 2023 (29)	29	NA/82256	OR	1.34 [1.08 to 1.67]	5/NA	Random	85%; < 0.0001	0.451	8	Very low
AR children	Infection within 2 years of age	high vs. low or none	Chen 2023 (29)	29	NA/56909	OR	1.25 [1.12 to 1.40]	11/NA	Random	89%; < 0.00001	0.451	8	Very low
AR children	Childhood acid-suppressant use	Any vs. none	Song 2022 (36)	36	NA/950640	HR	1.40 [1.24 to 1.58]	2/0/2/0	Random	97%; < 0.00001	NA	10	Very low
AR children	Prenatal acetaminophen exposure	Any vs. none	Zeng 2020 (43)	43	NA/1016	OR	2.42 [1.01 to 5.80]	1/0/1/0	Random	NA	NA	8	Very low
AR children	Acetaminophen exposure in early life	Any vs. none	Zeng 2020 (43)	43	NA/514729	OR	1.34 [1.21 to 1.49]	11/0/3/8	Random	90%; < 0.00001	NA	8	Very low
AR children	Birth order	≥2 vs. 1	Lisik 2023 (45)	45	NA/292067	RR	0.79 [0.73 to 0.86]	24/0/10/14	Random	86.09%;NA	NA	7	Very low
AR children	Sibship	≥2 vs. 1	Lisik 2023 (45)	45	NA/133926	RR	0.89 [0.83 to 0.95]	11/NA	Random	73.7%;NA	NA	7	Very low
AR children	Diagnosis of ADHD	With vs. without	Wang 2024 (40)	40	NA/397799	OR	1.78 [1.51 to 2.09]	10/0/2/8	Random	93%; <0.01	0.5565	9	Very low
AR children	Clinically diagnosed with ASD	With vs. without	Miyazaki 2015 (41)	41	2592/12277	OR	1.66 [1.49 to 1.85]	5/0/2/3	Random	0%; 0.46	NA	7	Very low
AR children	The association of KD	With vs. without	Lei 2021	42	NA/1220940	OR	1.726 [1.291 to 2.307]	4/0/0/4	Random	89%; 0.001	0.203	8	Very low
AR children	PNMS	Any vs. none	Flanigan 2018 (30)	30	NA	OR	1.36 [1.08 to 1.71]	3/0/2/1	Random	43.7%; NA	0.493	8	Very low
AR children	Cesarean section	With vs. without	Liu 2023 (31)	31	NA/1464868	OR	1.19 [1.12 to 1.27]	22/0/11/11	Random	68%; < 0.00001	0.137	9	Very low
Non-significant associations													
AR children	Childhood type 1 diabetes	With vs. without	Cardwell 2021	46	4231/77039	OR	0.97 [0.82 to 1.16]	18/NA	Random	56.6%;0.002	NA	7	Very low
AR children	Exposure to outdoor PM_2.5_ during pregnancy	Highest vs. lowest	Ai 2024 (47)	47	56091/145393	OR	1.61 [0.80 to 2.35]	8/0/6/2	Random	97%; < 0.01	<0.001	9	Very low
AR children	Exposure to outdoor PM_10_ during pregnancy	Highest vs. lowest	Ai 2024 (47)	47	5942/25238	OR	1.08 [0.84 to 1.38]	8/0/5/3	Random	95%; < 0.01	<0.001	9	Very low
AR children	Exposure to outdoor NO_2_ during pregnancy	Highest vs. lowest	Ai 2024 (47)	47	6215/25246	OR	0.99 [0.83 to 1.17]	8/0/6/2	Random	87%; < 0.01	<0.001	9	Very low
AR children	Prenatal vitamin D supplementation	Highest vs. lowest	Tareke 2020 (48)	48	685/1355	RR	1.03 [0.69 to 1.54]	3/3/0/0	Random	43%; 0.17	NA	9	Very low
AR children	Prenatal heavy metal exposure-CD	Highest vs. lowest	Chen 2024 (49)	49	NA/81920	RR	1.10 [0.93 to 1.29]	5/0/5/0	Fixed	0%; 0.66	NA	9	Very low
AR children	Prenatal heavy metal exposure-Pb	Highest vs. lowest	Chen 2024 (49)	49	NA/81920	RR	1.02 [0.68 to 1.54]	5/0/5/0	Random	83%; 0.00001	NA	9	Very low
AR Chinese children	O_3_	Highest vs. lowest	Zhang 2020	38	NA/15668	OR	0.98 [0.67 to 1.41]	3/0/2/1	Random	53%; 0.12	NA	8	Very low
AR children	Phthalates exposure in children’s urine	Highest vs. lowest	Oh 2024 (39)	39	101/3176	OR	1.094 [0.990 to 1.208]	6/0/0/6	Random	0%; 0.92	0.96772	8	Very low
AR children	Phthalate metabolites exposure in children’s urine	Highest vs. lowest	Oh 2024 (39)	39	546/6534	OR	1.028 [0.976 to 1.082]	11/0/1/10	Random	34.77%; 0.001	0.23811	8	Very low
AR children	Phthalates exposure in household dust	Highest vs. lowest	Oh 2024 (39)	39	474/1419	OR	1.021 [0.980 to 1.065]	6/0/0/6	Random	37.43%; 0.006	0.00004	8	Very low
AR 9–12 y	Residential greenness	Highest vs. lowest	Lambert 2017 (50)	50	NA/16194	OR	0.99 [0.87 to 1.12]	2/0/1/1	Random	72.9%; 0.002	NA	8	Very low
AR children	Duration of breastfeeding	<4 months vs. 4–6 months vs. ≥6 months vs. Unclear	Ding 2024 (51)	51	NA	OR	1.00 [0.91 to 1.10]	22/NA	Random	91.0%; < 0.001	>0.05	8	Very low
AR children	Breastfeeding pattern	Non vs. Partial vs. Exclusive vs. Unclear	Ding 2024 (51)	51	NA	OR	1.06 [0.96 to 1.6]	16//NA	Random	89.5%; < 0.001	>0.05	8	Very low
AR children	Serum 25(OH)D level	serum 25(OH)D ≧75 nmol/L vs. <50 nmol/L	Aryan 2017 (52)	52	NA/25965	OR	0.91 [0.81 to 1.01]	5/0/3/2	Random	74.9%; < 0.001	0.18	8	Very low
AR Newborn infant	Neonatal jaundice	With vs. without	Kuniyoshi 2020	53	NA/12213	OR	3.01 [0.88 to 10.30]	3/0/2/1	Random	93%; 0.01	NA	9	Very low
AR Newborn infant	Neonatal phototherapy	With vs. without	Kuniyoshi 2020	53	NA/94118	OR	1.38 [0.93 to 2.04]	2/0/2/0	Random	56%; 0.13	NA	9	Very low
AR children aged >2 y	Infant Formulas	PHF vs. CMF	Li 2024 (54)	54	NA/2941	RR	0.93 [0.81 to 1.08]	4/4/0/0	Random	0%; 0.95	0.013	10	Very low
AR children	Pet ownership in the first 2 years of life	Cat only vs. no pet ownership	Lodrup Carlsen 2012 (55)	55	NA/10384	OR	1.02 [0.80 to 1.30]	10/0/10/0	Random	0%; < 0.47	NA	6	Very low
AR children	Pet ownership in the first 2 years of life	Dog only vs. no pet ownership	Lodrup Carlsen 2012 (55)	55	NA/10107	OR	0.77 [0.55 to 1.07]	10/0/10/0	Random	0%; < 0.48	NA	6	Very low
AR children	Pet ownership in the first 2 years of life	Cat and dog only vs. no pet ownership	Lodrup Carlsen 2012 (55)	55	NA/7233	OR	0.79 [0.43 to 1.43]	10/0/10/0	Random	0%; < 0.49	NA	6	Very low
AR children	Pet ownership in the first 2 years of life	Bird only vs. no pet ownership	Lodrup Carlsen 2012 (55)	55	NA/10207	OR	1.28 [0.91 to 1.80]	10/0/10/0	Random	5%; < 0.39	NA	6	Very low
AR children	Pet ownership in the first 2 years of life	Rodent only vs. no pet ownership	Lodrup Carlsen 2012 (55)	55	NA/8648	OR	0.83 [0.48 to 1.45]	10/0/10/0	Random	0%; < 0.94	NA	6	Very low
AR and/or conjunctivitis	The introduction of CM or CMF	<4m vs. ≧4m	Yuan 2019	56	NA/2552	OR	1.05 [0.80 to 1.37]	3/0/3/0	Random	0%; NA	NA	8	Very low
AR/Hay fever 12 to 36 months	Maternal prenatal and/or postnatal n-3 LCPUFA supplementation	supplementation vs. placebo or no	Gunaratne 2015 (57)	57	28/833	RR	0.53 [0.25 to 1.12]	2/NA	Fixed	0%; 0.36	NA	9	Very low
AR/Hay fever beyond 36 months	Maternal prenatal and/or postnatal n-3 LCPUFA supplementation	supplementation vs. placebo or no	Gunaratne 2015 (57)	57	223/1392	RR	1.03 [0.81 to 1.3]	2/NA	Fixed	0%; 0.62	NA	9	Very low
AR children 0-5y	Vitamin D supplementation	high vs. low or none	Luo 2022 (58)	58	274/1676	RR	0.93 [0.78 to 1.11]	3/3/0/0	Fixed	47%; 0.15	NA	9	Very low
AR children	Fish consumption during pregnancy	high vs. low or none	Malmir 2016	59	NA	RR	0.91 [0.75 to 1.09]	NA	Random	0%; 0.324	NA	6	Very low
AR children	Maternal fish intake during pregnancy	high vs. low or none	Zhang 2016	28	NA/32589	RR	0.95 [0.62 to 1.45]	3/0/3/0	Random	44%; 0.81	NA	8	Very low
AR children over 5 years	Omega-3 supplementation during pregnancy	Highest vs. lowest	Venter 2022	60	193/1412	OR	0.76 [0.56 to 1.04]	2/0/0/2	Random	0%; 0.622	NA	7	Very low
AR children	Nutrients intake during pregnancy-Vitamin A	Highest vs. lowest	Feng 2023 (61)	61	NA	RR	1.05 [0.92 to 1.19]	NA	Random	NA	NA	8	Very low
AR children	Nutrients intake during pregnancy-Vitamin C	Highest vs. lowest	Feng 2023 (61)	61	NA	RR	0.93 [0.82 to 1.07]	NA	Random	NA	NA	8	Very low
AR children	Nutrients intake during pregnancy-Vitamin D	Highest vs. lowest	Feng 2023 (61)	61	NA	RR	1.03 [0.86 to 1.23]	NA	Random	0%; 0.562	NA	8	Very low
AR children	Nutrients intake during pregnancy-Vitamin E	Highest vs. lowest	Feng 2023 (61)	61	NA	RR	0.99 [0.83 to 1.18]	NA	Random	NA	NA	8	Very low
AR children	Nutrients intake during pregnancy-Folic acid	Highest vs. lowest	Feng 2023 (61)	61	NA	RR	0.95 [0.79 to 1.15]	NA	Random	NA	NA	8	Very low
AR children	Nutrients intake during pregnancy-Selenium	Highest vs. lowest	Feng 2023 (61)	61	NA	RR	0.99 [0.80 to 1.22]	NA	Random	NA	NA	8	Very low
AR children	Nutrients intake during pregnancy-Magnesium	Highest vs. lowest	Feng 2023 (61)	61	NA	RR	0.95 [0.76 to 1.19]	NA	Random	NA	NA	8	Very low
AR children	Nutrients intake during pregnancy-Zinc	Highest vs. lowest	Feng 2023 (61)	61	NA	RR	1.08 [0.86 to 1.35]	NA	Random	NA	NA	8	Very low
AR children	Nutrients intake during pregnancy-Fruit and vegetables	Highest vs. lowest	Feng 2023 (61)	61	NA	RR	1.01 [0.80 to 1.28]	NA	Random	0%; 0.615	NA	8	Very low
AR children	Nutrients intake during pregnancy-Cereals	Highest vs. lowest	Feng 2023 (61)	61	NA	RR	0.89 [0.62 to 1.28]	NA	Random	NA	NA	8	Very low
AR children	BCG Vaccination in Early Childhood	With vs. without	Zhao 2021 (62)	62	43823/69360	OR	0.87 [0.77 to 0.99]	12/2/0/10	Random	88%; 0.16	0.074	8	Very low
AR children	Early life natural exposure to n–6/n-3 PUFA ratio	Highest vs. lowest	Wu 2018	63	NA/2441	OR	1.08 [0.94 to 1.23]	2/0/2/0	Random	57.7%; 0.094	NA	9	Very low
AR children	Helminth infections	With vs. without	Arrais 2021	64	NA/100564	OR	0.96 [0.90 to 1.01]	48/NA	Random	NA	NA	6	Very low
AR children	Obese	With vs. without	Yeo 2024	65	NA/1703348	OR	1.02 [0.98 to 1.06]	11/0/1/10	Random	40%;0.08	0.89	9	Very low
AR children	Overweight	With vs. without	Yeo 2024	65	NA/1694396	OR	1.03 [0.99 to 1.07]	13/0/2/11	Random	45%;0.04	0.43	9	Very low
AR children	Underweight	With vs. without	Yeo 2024	65	NA/1602740	OR	1.02 [0.91 to 1.13]	6/0/2/4	Random	94%; < 0.01	NA	9	Very low

AR, allergic rhinitis; ADHD, attention deficit hyperactivity disorder; ASD, autism spectrum disorder; KD, Kawasaki disease; PNMS, Prenatal maternal psychosocial stress; OCP, oral contraceptive pill; PHF, partially hydrolyzed formula; CMF, cow’s milk formula; CM, cow’s milk; LCPUFA, long chain polyunsaturated fatty acids; BCG, Bacille Calmette–Guerin; PUFA, polyunsaturated fatty acids; T, total No. of studies; R, randomized controlled trials; C, cohort studies; P, population-based case-control and/or cross-sectional studies; AMSTAR, a measurement tool to assess systematic reviews; GRADE, Grading of Recommendations Assessment, Development, and Evaluation; NA, not available.

### 3.2 Diet and nutrition

This study identified three dietary and nutritional factors that play a protective role in the occurrence of AR in children. A meta-analysis pointed out that raw milk consumption in the first years of life (OR 0.68, 95% CI 0.57-0.82) and current raw milk consumption (OR 0.68, 95% CI 0.54-0.85) can significantly reduce the occurrence of AR in children (26). A meta-analysis of three cohort studies indicated that fish introduction between 6 and 12 months of age (OR 0.59, 95% CI 0.40-0.87) played a significant protective role in the occurrence of AR in children younger than 4 years (27).

### 3.3 Prenatal, gestational and postnatal factors

This study identified eight fetal and obstetric factors that significantly affect the risk of AR in children with AR. Among them, one factor played a protective role. High intake of fish during pregnancy or infancy could significantly reduce the risk of AR in children (RR 0.54, 95% CI 0.36-0.81) (28). Furthermore, relevant meta-analyses have found that infection during pregnancy (OR 1.34, 95% CI 1.08-1.67) (29), infection within the first 2 years of life (OR 1.34) 95% CI 1.08-1.67) (29), prenatal maternal psychosocial stress (RR 1.36, 95% CI 1.08-1.71) (30), caesarean section (OR 1.19, 95% CI 1.12-1.27) (31), maternal OCP exposure (OR 1.34, 95% CI 1.07-1.68) (32), prenatal smoke exposure (OR 1.12, 95% CI 1.04-1.21) (33), postpartum smoke exposure (OR 1.19, 95% CI 1.03-1.39) (33), and maternal exposure to smoking during pregnancy (OR 1.13 95% CI 1.02-1.26 (34) all significantly increased the risk of AR in children (**Figure 2**).

### 3.4 Early life and/or drugs factors

This study found that infection within the first 2 years of age (OR 1.25, 95% CI 1.08-1.67), early-life antibiotic use (OR 3.38, 95% CI 3.21-3.56) (35), early-life antibiotic use (HR 5.71, 95% CI 5.54-5.88) (35) and childhood acid-suppressant use (HR 1.40, 95% CI 1.24-1.58) (36) were significantly associated with an increased risk of AR in children. Furthermore, a meta-analysis of four cohort studies indicated that compared with non-food sensitization in early life, early life food sensitization significantly increased the prevalence of AR in children aged 2–12 years (OR 3.06, 95% CI 1.90-4.94) (37).

### 3.5 Environmental exposure

This study identified five environmental exposure factors that significantly affect the risk of AR in children with AR. A meta-analysis indicates that exposure to NO_2_ (OR 1.11, 95% CI 1.05-1.18) (38), SO_2_ (OR 1.03, 95% CI 1.01-1.05) (38), PM_10_ (OR 1.02, 95% CI 1.01-1.05) (38), and PM_2.5_ (OR 1.15, 95% CI 1.03-1.29) (38) significantly increased the incidence of AR in children. A meta-analysis of three cohort studies indicates exposure to phthalates and their metabolites in maternal urine also significantly increased the incidence of AR in children (OR 1.041, 95% CI 1.003-1.081) (39).

### 3.6 Comorbidity

This study found that a diagnosis of ADHD (OR 1.78, 95% CI 1.51-2.09) (40), a clinical diagnosis of ASD (OR 1.66, 95% CI 1.49-1.85) (41), and Kawasaki disease (KD) (OR 1.726, 95% CI 1.291-2.307) (42) were all significantly associated with an increased prevalence of AR in children.

### 3.7 Other outcome

This study also found that prenatal acetaminophen exposure (OR 2.42, 95% CI 1.01-5.80) (43) and acetaminophen exposure in early life (OR 1.34, 95% CI 1.21-1.49) (43) significantly increased the occurrence of AR in children. It is notable that a meta-analysis of eight cohort studies indicated that exposure to pets (OR 0.25, 95% CI 0.12-0.39) (44) could significantly reduce the occurrence of AR in children. Furthermore, birth order ≥2 (OR 0.79, 95% CI 0.73-0.86) (45) and having more than 2 siblings (OR 0.90, 95% CI 0.78-1.03) (45) were identified as protective factors associated with a reduced risk of AR in children.

### 3.8 Factors with insufficient evidence

It is particularly important to note that the aforementioned factors extracted in this study did not show statistically significant correlations in the combined analysis. This does not mean that there is “no association” or that these factors have “no effect” between them and the occurrence of childhood AR. The fundamental reason for this is that the number of included studies supporting these conclusions is extremely limited (most factors have fewer than 3 original studies or meta-analyses included), and some studies have small sample sizes, resulting in a severely insufficient overall statistical test power (Power), making it difficult to detect potential weak effects or accurately estimate their true association strength. A total of 46 risk factors that had insufficient evidence to determine an association with the occurrence of AR in children were extracted in this study, including childhood type 1 diabetes (46), exposure to outdoor PM_2.5_, PM_10_ and NO_2_ during pregnancy (47), prenatal vitamin D supplementation (48), prenatal heavy metal exposure to CD and Pb (49), O_3_ (38), phthalates exposure in children’s urine (39), phthalate metabolites exposure in children’s urine (39), phthalates exposure in household dust (39), residential greenness (50), duration of breastfeeding (51), breastfeeding pattern (51), serum 25(OH)D level (52), neonatal jaundice (53), neonatal phototherapy (53), infant formulas (54), pet ownership in the first 2 years of life (55), the introduction of CM or CMF (56), maternal prenatal and/or postnatal n-3 LCPUFA supplementation (57), vitamin D supplementation (58), fish consumption during pregnancy (59), maternal fish intake during pregnancy (28), omega-3 supplementation during pregnancy (60), nutrients intake during pregnancy-Vitamin A (61), Vitamin C (61), Vitamin D (61), Vitamin E (61), Folic acid (61), Selenium (61), Magnesium (61), Zinc (61), Fruit and vegetables (61), Cereals (61), BCG Vaccination in Early Childhood (62), early life natural exposure to n–6/n-3 PUFA ratio (63), helminth infection (64), obesity (65), overweight (65) and underweight (65). At present, there is no conclusive evidence to show that these factors are significantly associated with the occurrence of AR in children (**Table 1**).

### 3.9 Heterogeneity

Among the outcome measures included in this study, 65 studies reported heterogeneity, among which 34 had an I2 greater than 50%, including a diagnosis of ADHD (I^2^ = 93%) (40), KD (I^2^ = 89%) (42), exposure to pets (I^2^ = 99.8%) (44), and exposure to outdoor PM2.5 during pregnancy (I^2^ = 97%) (47), etc. There were 31 items with I^2^ values below 50%, including PNMS (I^2^ = 43.7%) (30), raw milk consumption in the first years of life (I^2^ = 0%) (26), and obesity (I^2^ = 40%) (65). The heterogeneity of the studies included in this article may mainly stem from the following reasons: differences in research design (such as the mixture of cross-sectional studies and cohort studies), distinct population characteristics (such as age stratification, region, race, and socioeconomic status), inconsistent definitions and measurement methods of exposure factors (such as environmental exposure assessment methods and diagnostic criteria), uneven control of confounding variables, and diversity in statistical models and adjustment strategies.

### 3.10 Publication bias

Publication bias was assessed for meta-analyses that included more than 10 individual studies, using funnel plots along with Egger’s and Begg’s statistical tests. In this study, 42 outcome measures showed evidence of publication bias, and 8 meta-analyses demonstrated statistical evidence of potential publication bias (Egger’s or Begg’s test P < 0.05), including childhood type 1 diabetes (46), exposure to outdoor PM_2.5_ during pregnancy (47), exposure to outdoor PM_10_ during pregnancy (47), exposure to outdoor NO_2_ during pregnancy (47), neonatal jaundice (53), neonatal phototherapy (53), infant formula use (54), and phthalate exposure in household dust (39). The remaining 34 items did not show significant small-study effects. For the majority of risk factors, however, the number of underlying primary studies within each meta-analysis was fewer than 10; therefore, formal statistical testing for publication bias was not conducted for these outcomes, which represents a methodological limitation. Overall, while detectable publication bias appeared limited among the larger meta-analyses, the possibility of unpublished null or negative findings affecting the broader evidence base cannot be ruled out, particularly for less frequently studied risk factors.

## 4 Discussion

With the changes in living environment and the acceleration of urbanization, allergic rhinitis has become a highly prevalent chronic inflammatory disease worldwide, especially among children. Its characteristic symptoms such as nasal itching, sneezing, runny nose, and nasal congestion, not only directly affect the quality of life of pediatric patients, but are also closely related to comorbidities such as sleep disorders, decreased attention, impaired learning ability, and asthma. In the long term, it poses a significant burden on both personal health and the socioeconomic system. The pathogenesis of AR is complex, involving interactions among multiple factors, including genetic susceptibility, environmental exposure, early-life immune programming, and epigenetic regulation, presenting a distinct “developmental origins” feature. Although multiple potential risk factors, including genetic background, caesarean section, early microbial exposure, air pollution, and smoking during pregnancy, have been identified, the existing evidence still demonstrates fragmentation, substantial heterogeneity, and low overall quality. Many reported associations have not yet reached consistent conclusions or had their underlying mechanisms clearly elucidated. Therefore, systematically integrating existing evidence, clarifying contradictory findings, and identifying key risk windows and modifiable factors are of critical importance for promoting a shift in AR prevention strategies from “symptom-based treatment” to “etiology-oriented prevention”. This study adopts an umbrella review approach to comprehensively summarize and evaluate factors influencing the occurrence of AR, providing an integrated scientific evidence base for developing targeted preventive strategies and improving long-term outcomes in children. It is important to note that the vast majority of associations identified in this umbrella review are supported by low or very low certainty evidence, primarily derived from observational studies. Therefore, these findings should be interpreted as statistical associations rather than established causal relationships. The Bradford Hill criteria for causation, including consistency, temporality, and biological gradient, have not been systematically satisfied for most risk factors examined.

### 4.1 Multidimensional influencing factors of childhood AR onset

We found that consuming raw milk and fish, or a high intake of fish during pregnancy or infancy, suggested protective effects. This might be attributed to the fact that the immunomodulatory components retained in these natural foods (such as microbiota and omega-3 fatty acids) help promote the establishment of immune tolerance. Epidemiological evidence suggests that growing up on a farm is a protective factor against allergies, partly due to the consumption of raw milk. Raw milk components (e.g., TGF-β, lactoferrin, vitamin D, immunoglobulins, sialic acid oligosaccharides) may protect against respiratory infection and sensitization by strengthening epithelial barriers, directly neutralizing pathogens/allergens, and modulating systemic immunity via the gut-lung axis through regulatory T-cell and tolerogenic dendritic cell induction (66). It is critically important to note that current pediatric health authorities, including the American Academy of Pediatrics and the World Health Organization, do not recommend raw milk consumption due to well-documented risks of foodborne infections. The observed protective association from observational studies should not be interpreted as a recommendation for raw milk intake. Rather, this finding highlights the need to identify the specific immunomodulatory components in raw milk that could be safely incorporated into processed dairy products. Fish, as a food source, is rich in compounds with immunomodulatory properties, including omega-3 fatty acids, melatonin, tryptophan, taurine and polyamines. Notably, long-chain omega-3 fatty acids may exert anti-inflammatory effectsby altering the types of prostaglandins, leukotrienes and thromboxane produced by arachidonic acid (67). This mechanism is closely related to the pathophysiology of AR. These findings support an extension of the “hygiene hypothesis”, suggesting that early exposure to diverse microorganisms and antigens is beneficial for healthy immune development.

Prenatal, perinatal, and postnatal factors may significantly impact on the occurrence and development of this disease. The protective effect of high fish intake during pregnancy in mothers is consistent with the aforementioned findings, while caesarean section, maternal psychological stress during pregnancy, smoking (both active and passive), infection during pregnancy, and exposure to oral contraceptives (OCPs) are all potentially associated with an increased risk of AR in the offspring. Cesarean section may affect immune maturation by altering the colonization of the initial intestinal flora in newborns. Studies have shown that cesarean section is associated with lower abundances of tryptophan, bile acids, and phenylalanine metabolites, which significantly increase the risk of asthma in school-aged children by interfering with the colonization patterns of the dominant intestinal flora (i.e., Bifidobacterium and Bacteroides) after natural childbirth and disrupting the immune responses (68). The association between caesarean section and AR risk adds to the growing body of evidence supporting medically indicated rather than elective caesarean delivery, although causality remains to be established. Prenatal adverse exposures (tobacco smoke, stress) may exert long-term programming effects on fetal immune development via epigenetic modifications or inflammatory pathways; smoke directly induces nasal inflammation by altering neutrophil activation and epithelial permeability (69). Pre-pregnancy OCP use is associated with increased offspring respiratory allergy within 5 years (70). Mechanistically, interactions among GATA3 variants, OCP exposure and DNA methylation may drive epigenetic changes (71), with Developmental Origins of Health and Disease (DOHaD) theory suggesting possible germline transmission (72, 73); however, direct evidence for gametophyte epigenetic effects of OCP is currently absent. It is worth noting that these mechanistic explanations remain speculative and require confirmation in well-designed experimental studies.

Among early life or drug factors, infantile infections, antibiotic use, acid suppressant use, and early food sensitization are all closely related to an increased risk of AR. This highlights that the first few years of life are a critical window for immune programming. Frequent infections and drug interventions (especially antibiotics) may disrupt the microbiota-immune axis balance and increase susceptibility to atopic diseases. Animal studies have shown that antacids can inhibit the decomposition of ingested proteins and promote the production of IgE antibodies (74). Gayen et al.’s research indicates that in neonates exposed to fetal membrane infection during pregnancy, the control of inflammatory, immune and respiratory processes is influenced by the differential gene expression in white blood cells (75). In animal experiments, elevated IL-17A was associated with allergic airway inflammation in neonates infected with Streptococcus pneumoniae (76). The latest research also indicates that mice infected with Streptococcus pneumoniae exhibit more pronounced airway responses, with higher levels of specific IgE and Th2 cytokines in lung serum (77).

In terms of environmental exposure, outdoor air pollutants (NO, SO, and PM) and exposure to phthalates during pregnancy have been suggested as risk factors. The relationship between environmental exposure and rhinitis and asthma has been widely documented (78, 79). Air pollution includes both atmospheric particulate matter (PM) and gaseous pollutants. The relationship between PM and asthma/rhinitis has aroused considerable interest (80). Recently, research on ultrafine particulate matter (UFP) has increased. Due to its small particle size, UFP can directly reach the alveoli. Reports increased that nitrogen dioxide, as a transportation-related pollutant, is associated with asthma (81). These findings expand the scope of AR prevention from traditional allergens to a broader range of environmental toxins, highlighting the importance of air quality management and reducing exposure to plasticizers in public health prevention. It is worth noting that some non-traditional protective factors are considered potential, such as contact with pets, later birth order, and having more siblings. This further supports the “microbial exposure” and “sibling effect” hypotheses, that is, early and diverse microbial environmental exposure helps to reduce the risk of allergies.

Comorbidity analysis indicated that AR was significantly associated with attention deficit hyperactivity disorder (ADHD), autism spectrum disorder (ASD), and Kawasaki disease (KD). This suggests that common underlying pathophysiological mechanisms, such as systemic inflammation or abnormal immune regulation, may simultaneously act on the respiratory tract, nervous system, and cardiovascular system. Researchers believe that a series of events triggered by various stimuli (i.e., environmental factors, food, stress, or infection, and atopic diseases) may initiate an immune inflammatory cascade reaction, thereby leading to neuroimmune inflammation with clinical manifestations of ADHD (82). ADHD and AR may share a common mechanism and represent a comorbidity that links the nervous system to the immune system. As early as 2013, a study pointed out that 47.6% of children with autism have allergic manifestations (bronchial asthma, atopic dermatitis, and/or AR), and allergies may be a contributing factor to the elevated levels of brain-specific autoantibodies in some children with autism (83). Dysbiosis-related Treg (regulatory)/T helper type (Th)17 imbalance and type 2 inflammation may explain part of the immunopathogenic connection between KD and allergic diseases (84, 85). Liew et al. identified that KD children without CAL have an intensified form of AR and any other allergies when compared with controls, consistent with lower Th2 cytokines in KD patients with CAL (86, 87). However, one study suggests that although an increased risk of asthma and AR was observed after KD, it remains unclear whether this is attributable to aspirin treatment (42).

### 4.2 Potential stratified prevention strategies

The results of this study emphasize the significance of early-life intervention, pregnancy management, environmental regulation, and identification of high-risk children in reducing the incidence of AR in children. It highlights that understanding the risk of AR in children must shift from a single, static listing of factors to a dynamic and interactive life-course model, with the aim of providing guidance for clinical practice and public health management in the precise prevention of AR in children. Considering the risk factors that have been reported, an exploratory hypothesis generation scheme may be considered for AR in children. First, high-priority intervention targets include discouraging smoking during pregnancy and after childbirth, avoiding non-medically indicated caesarean sections, reducing the use of non-essential antibiotics and acid suppressants in early life, and encouraging the introduction of diverse complementary foods, such as fish, at appropriate ages. It is worth noting that decisions should be made cautiously based on individual circumstances. The decision regarding pet ownership should be made on an individual basis, weighing potential allergenic risks against possible immunological benefits, and current evidence does not support a universal recommendation for or against pet exposure for allergy prevention. Second, for targets requiring individualized assessment, the evidence may be contradictory or the underlying mechanisms complex, such as psychological stress during pregnancy and exposure to air pollution, for which consultation and supportive interventions may be considered. Finally, future research should continue to focus on emerging directions, particularly the role of the intestinal microbiota, supplementation with specific nutrients (such as n-3 PUFA) during pregnancy, and the control of exposure to environmental endocrine disruptors (such as phthalates). It should be noted that this framework is proposed as a hypothesis-generating scheme rather than clinical guidance, and its components require validation through prospective cohort studies and, where feasible, randomized controlled trials before any clinical implementation can be considered.

### 4.3 Immunological mechanisms linking risk factors to AR in children

Although the causal nature of many identified risk factors remains to be firmly established, a growing body of mechanistic studies suggests that these diverse exposures may converge on several shared immunological pathways that ultimately predispose children to AR. Synthesizing the available evidence from experimental, clinical, and microbiome studies, we summarize the major mechanisms potentially linking these risk factors to the pathogenesis of childhood AR as follows: (1) Impaired epithelial barrier function: Environmental pollutants (e.g., PM2.5, NO2) and tobacco smoke can disrupt the epithelial barrier function of the nasal mucosa, leading to an increase in the permeability of allergens and the activation of antigen - presenting cells. (2) Th2 immune polarization: Early use of antibiotics and cesarean section may interfere with the colonization of the intestinal microbiota, promoting Th2 - type immune deviation and enhancing the susceptibility to IgE - mediated allergic reactions. (3) Imbalance of regulatory T cells (Treg): Components such as TGF - β and lactoferrin in raw milk may maintain immune tolerance by facilitating Treg differentiation. In contrast, early infections and inflammation may disrupt the balance of Treg/Th17. (4) Innate lymphoid cells (ILC2): Upon allergen exposure, ILC2 rapidly secrete IL - 5 and IL - 13, serving as an early initiator of the type 2 immune response. Air pollutants can directly stimulate airway epithelial cells to secrete IL - 25, IL - 33, and TSLP, thereby activating ILC2. (5) Microbiota - immune interaction: The composition of the microbiota in the gut and nasal cavity impacts local and systemic immune development. Antibiotics, cesarean section, and dietary factors can influence the risk of allergies by modifying the composition of the microbiota. (6) Association between environmental exposure and immune maturation: Farm environments and contact with pets may promote the normal maturation of the immune system by increasing microbial diversity, which is consistent with the modern understanding of the “hygiene hypothesis” and the “microbial exposure hypothesis”.

### 4.4 Quality of evidence, heterogeneity of studies, and methodological limitations

This study reveals that there is significant heterogeneity and inconsistency in the current evidence, with pet exposure being the most typical example. Early studies mostly reported its harmful effects, while recent large-scale analyses suggest that it has a protective effect. This inconsistency may stem from the evolution of research design (such as the refinement of exposure time windows, pet types, and confounding factor control), differences in population genetic background and lifestyle, as well as publication bias. For example, for ADHD (I² = 93%), we point out that the heterogeneity mainly stems from differences in research design (a mixture of cross-sectional studies and cohort studies) and the different diagnostic criteria for ADHD; for pet exposure (I² = 99.8%), the heterogeneity may arise from differences in stratification of pet type, exposure time window, and family history of allergies; for Kawasaki disease (I² = 89%), the heterogeneity may be related to the evolution of KD treatment regimens and the duration of follow-up. This highlights the need for caution when interpreting the conclusions of single studies or early meta-analyses and emphasizes the necessity of conducting regular evidence updates and re-evaluations. Furthermore, given that most included meta-analyses contained fewer than 10 primary studies, the statistical power to detect publication bias was limited, and the absence of significant Egger test results should not be interpreted as evidence of no publication bias. It should be noted that AMSTAR assesses the methodological quality of the meta-analysis itself, while GRADE assesses the overall certainty of the evidence. Therefore, even if a meta-analysis receives a high score on AMSTAR (such as 10 points), if the original studies included in it are mostly of observational design and have a risk of bias, the GRADE score will still be very low. This difference precisely reflects a core dilemma in the current field: even with high-quality secondary research, the inherent limitations of the original study design cannot be compensated for.

This study systematically retrieved four mainstream English biomedical databases: PubMed, Embase, Web of Science, and the Cochrane Library. Failure to cover specialized databases in specific fields (such as Scopus and CINAHL), regional databases, or academic resource libraries in non-English languages (such as Chinese and Spanish) may lead to potential language and regional biases, which limit the generalizability of the research conclusions across different populations worldwide. More importantly, our search relied on published academic literature with full text available, without systematically incorporating grey literature such as dissertations, conference abstracts, unpublished results from clinical trial registration platforms, or government and institutional reports, making it more likely that positive or statistically significant studies were published and included in the analysis. This may lead to a systematic overestimation of the effect sizes of risk factors. Furthermore, the majority of the evidence synthesized in this study was derived from observational studies (such as cross-sectional and cohort studies), making it difficult to fully control for residual confounding and reverse causation. This implies that the findings of this study primarily reflect statistical associations rather than definitive causal relationships. Finally, medical diagnostic criteria and clinical practice standards are subject to continuous evolution. Temporal and regional variability in the diagnosis and definition of AR and its risk factors across different studies may affect the applicability of the findings to current clinical practice.

## 5 Conclusion

This study emphasizes prenatal, gestational, and postnatal factors, environmental exposure, early-life and/or drug-related factors, and comorbidities as the main risk factors for allergic rhinitis in children. In contrast, exposure to pets, raw milk consumption in the first years of life, current raw milk consumption, fish introduction between 6 and 12 months of age, high intake of fish during pregnancy or infancy, birth order, and sibship are protective factors for AR in children. Despite the abundance of available meta-analyses, the overall quality of the evidence remains limited. It is critical to note that the overwhelming majority of the synthesized evidence originates from observational studies with low to very low certainty, precluding any inference of causality, with only one rated as moderate. These findings should be interpreted as hypothesis-generating associations rather than established causal relationships. Moreover, there is widespread heterogeneity, reflecting differences in study design, population, and exposure assessment. To advance the field, future research should prioritize prospective cohort studies with standardized exposure definitions, rigorous confounding control, and transparent reporting. Based on the current evidence map, several modifiable factors emerge as promising targets for future preventive interventions in childhood AR, but their implementation requires confirmation through prospective cohort studies and, where feasible, randomized controlled trials. Clinical recommendations should await higher-certainty evidence.

## Data Availability

The original contributions presented in the study are included in the article/**Supplementary Material**. Further inquiries can be directed to the corresponding authors.
