# Supplementary material for: Risk factors for allergic rhinitis in children: an umbrella review of systematic review and meta-analysis
Source: Front Immunol. 2026 Sep 7;17:1849271. doi: 10.3389/fimmu.2026.1849271 (PMC13590725; doi:10.3389/fimmu.2026.1849271)
Supplement: Supplementary file 1 [file Table1.docx]

**Table S1 Literature search strategy**

Pubmed-597

((("Risk Factors"[Mesh]) OR ((((((((((((Risk Factor) OR (Population at Risk)) OR (Populations at Risk)) OR (Risk Scores)) OR (Risk Score)) OR (Risk Factor Scores)) OR (Risk Factor Score)) OR (Health Correlates)) OR (Social Risk Factors)) OR (Social Risk Factor)) OR (Risk)) OR (Incidence))) AND (("Rhinitis, Allergic"[Mesh]) OR ((Allergic Rhinitides) OR (Allergic Rhinitis)))) AND (systematic review OR meta-analysis)

Embase-904

((Risk Factors or (Risk Factor or Population at Risk or Populations at Risk or Risk Scores or Risk Score or Risk Factor Scores or Risk Factor Score or Health Correlates or Social Risk Factors or Social Risk Factor or Risk or Incidence)) and (Rhinitis, Allergic or (Allergic Rhinitides or Allergic Rhinitis)) and (systematic review or meta-analysis)).af.

Cochrane-159

((Risk Factors or (Risk Factor or Population at Risk or Populations at Risk or Risk Scores or Risk Score or Risk Factor Scores or Risk Factor Score or Health Correlates or Social Risk Factors or Social Risk Factor or Risk or Incidence)) and (Rhinitis, Allergic or (Allergic Rhinitides or Allergic Rhinitis)) and (systematic review or meta-analysis)).af.

WOS-600

(((Risk Factors) OR ((((((((((((Risk Factor) OR (Population at Risk)) OR (Populations at Risk)) OR (Risk Scores)) OR (Risk Score)) OR (Risk Factor Scores)) OR (Risk Factor Score)) OR (Health Correlates)) OR (Social Risk Factors)) OR (Social Risk Factor)) OR (Risk)) OR (Incidence))) AND ((Rhinitis, Allergic) OR ((Allergic Rhinitides) OR (Allergic Rhinitis)))) AND (systematic review OR meta-analysis) (Topic)
